# Genome-wide analysis of the potato *Hsp20* gene family: identification, genomic organization and expression profiles in response to heat stress

**DOI:** 10.1186/s12864-018-4443-1

**Published:** 2018-01-18

**Authors:** Peng Zhao, Dongdong Wang, Ruoqiu Wang, Nana Kong, Chao Zhang, Chenghui Yang, Wentao Wu, Haoli Ma, Qin Chen

**Affiliations:** 1 State Key Laboratory of Crop Stress Biology for Arid Areas, College of Agronomy, Northwest A & F University, Yangling, Shaanxi 712100 China; 2 Innovation Experimental College, Northwest A & F University, Yangling, Shaanxi 712100 China

**Keywords:** Genome-wide, Potato, *Hsp20* gene family, Heat stress

## Abstract

**Background:**

Heat shock proteins (Hsps) are essential components in plant tolerance mechanism under various abiotic stresses. Hsp20 is the major family of heat shock proteins, but little of Hsp20 family is known in potato (*Solanum tuberosum*), which is an important vegetable crop that is thermosensitive.

**Results:**

To reveal the mechanisms of potato Hsp20s coping with abiotic stresses, analyses of the potato *Hsp20* gene family were conducted using bioinformatics-based methods. In total, 48 putative potato *Hsp20* genes (*StHsp20s*) were identified and named according to their chromosomal locations. A sequence analysis revealed that most *StHsp20* genes (89.6%) possessed no, or only one, intron. A phylogenetic analysis indicated that all of the *StHsp20* genes, except 10, were grouped into 12 subfamilies. The 48 *StHsp20* genes were randomly distributed on 12 chromosomes. Nineteen tandem duplicated *StHsp20s* and one pair of segmental duplicated genes (*StHsp20-15* and *StHsp20-48*) were identified. A *cis*-element analysis inferred that *StHsp20*s, except for *StHsp20-41*, possessed at least one stress response *cis*-element. A heatmap of the *StHsp20* gene family showed that the genes, except for *StHsp20-2* and *StHsp20-45*, were expressed in various tissues and organs. Real-time quantitative PCR was used to detect the expression level of *StHsp20* genes and demonstrated that the genes responded to multiple abiotic stresses, such as heat, salt or drought stress. The relative expression levels of 14 *StHsp20* genes (*StHsp20-4*, *6*, *7*, *9*, *20*, *21*, *33*, *34*, *35*, *37*, *41*, *43*, *44* and *46*) were significantly up-regulated (more than 100-fold) under heat stress.

**Conclusions:**

These results provide valuable information for clarifying the evolutionary relationship of the *StHsp20* family and in aiding functional characterization of *StHsp20* genes in further research.

**Electronic supplementary material:**

The online version of this article (dio: 10.1186/s12864-018-4443-1) contains supplementary material, which is available to authorized users.

## Background

Plants live in an open environment and are exposed to various abiotic and biotic stresses. The increased temperatures associated with global warming have adverse effects on plant growth and development [1]. During tuber development, high temperatures can change plant source–sink relationship, which disrupts tuber initiation, and thus reduces yield and quality [2–4]. To survive and avoid adverse effects under high temperature, plants established self-defense mechanisms during evolution. Heat shock proteins (Hsps) are a group of proteins synthetized under high temperature that exist in living organisms from bacteria to humans [5]. In plants, the *Hsp* genes participate in many developmental processes and responding to abiotic stresses [6, 7].

According to previous studies, Hsps can be grouped into five families including Hsp100, Hsp90, Hsp70, Hsp60 and Hsp20 based on their molecular weight and sequence homology [6, 8]. The molecular weight of Hsp20 protein ranges from 15 to 42 kDa, thus is also called as small Hsp [9]. Hsp20 is the major family of heat shock proteins induced by elevated temperature-associated stress in plants [10, 11]. Hsp20 is encoded by a multigene family and is considered the most produced protein under heat stress conditions in many higher plants [12, 13].

Hsp20s are ATP-independent molecular chaperones and can form oligomeric protein complexes of 200–800 kDa, which consist of 9 to 50 subunits [14, 15]. Hsp20 can avert protein denaturation, and thus maintain the stability and normal functions of proteins in both eukaryotic and prokaryotic cells [6, 16]. The existing evidence suggests that Hsp20 plays an important role in plant heat tolerance. Hsp20s possess a conserved structure, consisting of a variable N-terminal region, a more conserved C-terminal region and a C-terminal extension [6]. The more conserved C-terminal region is usually named as the alpha-crystallin domain (ACD), which contains approximately 80 to 100 amino acid residues. The three different regions possess varied functions. The ACD functions in substrate interactions, while the N-terminal region participates in substrate binding and the C-terminal extension is responsible for homo-oligomerization [17–20]. The ACD contains two conserved regions, one in the N-terminal consensus region and the other is connected through a hydrophobic β6-loop at the C-terminal common region. The two conserved regions consist of 4 anti-parallel sheets and 3 β-strands respectively [16, 21]. Furthermore, unlike other *Hsp* families, the *Hsp20* gene family exhibits extensive sequence variability and evolutionary divergence [22].

The number of plant *Hsp20* genes is approximately four times greater than that of animals [10]. The *Hsp20* gene family members have been investigated in many plants, such as *Arabidopsis*, rice, soybean, pepper and tomato. There are 19 *Hsp20* genes in *Arabidopsis* [23], 39 in rice [24], 51 in soybean [25], 35 in pepper [26] and 42 in tomato [27]. Following maize, wheat and rice, potato is the fourth-largest food crop in the world. Potatoes are formed from underground stems through a process known as tuberization, but high temperatures inhibit the process and decrease the amount of photosynthetic product transported into the tubers, causing a large yield loss [28]. To date, the potato *Hsp20* gene family members have not been identified and their functions under heat stress conditions remain to be elucidated. With the availability of the whole-genome sequence of potato, it is now possible to more fully study the potato *Hsp20* gene family.

Here, we used bioinformatics methods to identify *Hsp20* genes from potato genome, and analyze the sequence features, chromosomal locations, phylogenetic relationships, *cis*-elements, tissue-specific expression levels and dynamic expression patterns in response to different abiotic stresses, including heat stress. The results provide useful information for further functional investigations of the *StHsp20* gene family.

## Methods

### Identification of the *Hsp20* family members in potato genome

The whole potato protein sequence was downloaded from the Potato Genome Sequencing Consortium (PGSC, http://potato.plantbiology.msu.edu/integrated_searches.shtml). To identify potato Hsp20 candidates, the Hidden Markov Model (HMM) analysis was used for the search. We downloaded HMM profile of Hsp20 (PF00011) from Pfam protein family database (http://pfam.xfam.org/) and used it as the query (*P* < 0.001) to search the potato protein sequence data [29]. To avoid missing probable Hsp20 members because of incomplete ACD domains, a BLASTP-algorithm based search using *Arabidopsis* Hsp20 amino acid sequences as queries was conducted with an e-value ≤1e^− 3^. Additionally, keywords “Hsp20” and “small heat shock protein” were employed to search against PGSC database. After removing all of the redundant sequences, the output putative Hsp20 protein sequences were submitted to CDD (https://www.ncbi.nlm.nih.gov/Structure/bwrpsb/bwrpsb.cgi), Pfam and SMART (http://smart.embl-heidelberg.de/) to confirm the conserved Hsp20 domain. The predicted protein sequences lacking the Hsp20 domain or with a molecular weight outside of the 15–42-kDa range were excluded. All of the non-redundant and high-confidence genes were assigned as potato *Hsp20*s (*StHsp20s*). These *StHsp20* genes were named on the basis of their positions on pseudomolecules [24].

### Sequence analysis and structural characterization

All of the high-confidence Hsp20 sequences were submitted to ExPASy (http://web.expasy.org/protparam/) to calculate the number of amino acids, molecular weights and theoretical isoelectric points (pI). The chromosomal locations and intron numbers of *StHsp20*s were acquired through the PGSC. The MEME program (version 4.11.2, http://alternate.meme-suite.org/tools/meme) was used to identify the conserved motifs in the StHsp20s sequences, with the following parameters: any number of repetitions, maximum of 10 misfits and an optimum motif width of 6 - 200 amino acid residues. The exon–intron structures of the *StHsp20* genes were identified on the Gene Structure Display Server (GSDS, http://gsds.cbi.pku.edu.cn/) [30].

### Chromosomal localization and gene duplication

The chromosomal positions of the *StHsp20* genes were acquired from the potato genome browser at the PGSC. MapChart software [31] was used for the mapping of *StHsp20* genes’ chromosomal positions and relative distances. The *StHsp20* gene duplication was confirmed based on two criteria: (a) the length of the shorter aligned sequence covered > 70% of the longer sequence; and (b) the similarity of the two aligned sequences were > 70% [32, 33]. Two genes separated by five or fewer genes in 100-kb chromosome fragment were considered as tandem duplicated genes [34]. The segmental duplicated genes of *StHsp20* were identified by searching the segmental genome duplications of potato at the Plant Genome Duplication Database (PGDD, http://chibba.agtec.uga.edu/duplication/).

### Phylogenetic analysis and classification of potato *Hsp20* genes

The full-length amino acid sequences of Hsp20s (Additional file 1: Table S1) derived from *Arabidopsis* [35], soybean [25], rice [24] and *Populus* [36] combined with newly identified StHsp20s were used for phylogenetic analysis. All of the acquired sequences were first aligned by ClustalX (version 1.83) software [37] with the default parameters. An unrooted neighbor-joining phylogenetic tree was constructed using MEGA6 software [38] with bootstrap test of 1000 times. The potato *Hsp20* genes were classified into different groups according to the topology of phylogenetic tree and the classifications of *Hsp20s* in four other species.

### Analysis of *Cis*-acting element in *StHsp20* genes’ promoters

The upstream sequences (1.5 kb) of the *StHsp20*-coding sequences were retrieved from the PGSC and then submitted to PlantCARE (http://bioinformatics.psb.ugent.be/webtools/plantcare/html/; [39]) to identify six regulatory elements, abscisic acid (ABA)-responsive elements, involved in the ABA responsiveness; dehydration-responsive elements (DREs), involved in dehydration, low-temp and salt stresses; heat stress elements (HSEs), involved in heat stress response; low temperature responsive elements (LTRE), involved in low-temperature response; TC-rich repeats, involved in defense and stress response; and W-boxes, binding site of WRKY transcription factor in defense responses.

### Plant materials and abiotic stress treatments

The doubled monoploid (DM) potato was used in this study. All of the lines were cultured in Murashige and Skoog (MS) medium [40] containing 3% sucrose and 0.8% agar at pH 5.9. The plant material was sustained in an artificial climate chamber with 16 h light/8 h dark photoperiod and temperature of 22 ± 1 °C. The four-week-old plantlets were then transferred into cuvettes containing 1/2 MS liquid medium and maintained in an artificial growth chamber at 22 ± 1 °C (16 h light/8 h dark period) for a week before being subjected to an abiotic stress. For heat stress, the plantlets were exposed to 35 °C; for salt stress, the plantlets were incubated with 150 mM NaCl; and for drought stress, the plantlets were treated with 260 mM mannitol. Under these different stress conditions, the aboveground of whole plants were collected at 0, 3 and 24 h after treatments. All of the collected samples were froze in liquid nitrogen rapidly and stored at − 80 °C refrigerator before RNA extraction.

### RNA-sequencing (RNA-seq) data analysis of *StHsp20* genes

The Illumina RNA-seq data were downloaded from the PGSC to study the expression patterns of *StHsp20* genes. The RNA-seq data (Additional file 2: Table S2) included various developmental stages, tissues and stress treatments. To render the data suitable for cluster displays, absolute FPKM values were divided by the mean of all of the values, and the ratios were transformed by log2. HemI [41] software was used to generate the heatmap.

### Total RNA extractions and expression analyses of potato *Hsp20* genes

Primer Premier 5 was used to design primers specific to the *StHsp20* genes (Additional file 3: Table S3). Total RNA was extracted using an RNAsimple Total RNA Kit (BioTeke, Beijing, China). The cDNA was reverse-transcribed by First Strand cDNA Synthesis Kit, ReverTra Ace-α (TOYOBO, Shanghai, China). All of the operational procedures followed the manufacturer’s protocols. Before the qRT-PCR analysis, 1 μl cDNA was diluted with 4 μl nuclease-free water.

qRT-PCR was carried out using the KAPA SYBR FAST qPCR Kit Master Mix (2×) Universal (KAPA BIOSYSTEMS, Boston, United States) on a Bio-Rad CFX96 Real Time PCR System. Each PCR reaction was conducted in a 20-μl reaction volume containing 10 μl KAPA SYBR, 0.5 μl 10 μM solution of each primer, 1 μl diluted cDNA and 8 μl ddH_2_O. The PCR program was set as follow: 95 °C for 2 min and 40 cycles of 95 °C for 5 s and 60 °C for 30 s. The melt curve was analyzed from 65 °C to 95 °C with increments of 0.5 °C every 5 s. For each sample, three biological repeats, with two technical replicates each, were performed to acquire reliable results. The housekeeping gene *ef1α* was used as the internal reference gene. The synthetic cDNA was diluted to 3-, 9-, 27- and 81-fold to establish the standard curve for each *StHsp20* gene and *ef1α*. The relative expression levels of the *StHsp20* genes were calculated using the standard curve and normalized by the control’s expression. The results were displayed by means ± standard deviation (SD).

## Results

### Identification and analysis of *StHsp20* genes

A total of 58 Hsp20s were obtained by HMM analysis, 52 sequences were found by local BLASTP, and 35 sequences were acquired by keyword search against the PGSC database. After removing the repetitive sequences, 65 sequences were reserved and submitted to CDD, Pfam and SMART to confirm the ACD domain. Sequences without a typical ACD domain and with a molecular weight outside of the 15–42-kDa range were excluded. Finally, 48 sequences were confirmed as potato *Hsp20* genes and named based on their chromosomal locations. Gene names, gene IDs, chromosomal locations, open reading frame lengths, exon numbers, amino acid numbers, molecular weights and pIs were listed in Table 1. The lengths of the StHsp20 proteins ranged from 133 (StHsp20-36) to 303 amino acids (StHsp20-15). The molecular weights of StHsp20s were between 15.3 kDa (StHsp20-36) and 34.0 kDa (StHsp20-15). *StHsp20* genes were distributed on 12 potato chromosomes. The predicted pI values of StHsp20 ranged from 4.91 (StHsp20-5) to 9.88 (StHsp20-39).

**Table 1:** Features of *StHsp20* genes identified in potato

Name	Gene ID	Chr.	Genomic Location	ORF	Exon	AA	MW (kDa)	pI
StHsp20-1	PGSC0003DMG400008713	1	6195606 - 6196992	732	2	243	26.8	5.80
StHsp20-2	PGSC0003DMG400008714	1	6199128 - 6200118	669	2	222	24.5	8.91
StHsp20-3	PGSC0003DMG400008715	1	6205818 - 6207478	639	2	212	23.8	9.30
StHsp20-4	PGSC0003DMG400020718	1	79702817 - 79703750	576	1	191	21.8	6.87
StHsp20-5	PGSC0003DMG400016460	2	36223235 - 36224237	414	2	137	15.7	4.91
StHsp20-6	PGSC0003DMG400012619	2	47595303 - 47596399	426	2	141	16.3	8.32
StHsp20-7	PGSC0003DMG400003219	3	46254502 - 46257461	702	2	233	25.9	6.98
StHsp20-8	PGSC0003DMG400025350	3	53138385 - 53139192	651	2	216	24.5	4.98
StHsp20-9	PGSC0003DMG400024476	3	53878612 - 53879610	576	1	191	21.9	6.87
StHsp20-10	PGSC0003DMG400009173	3	61718867 - 61719420	444	2	147	16.5	7.64
StHsp20-11	PGSC0003DMG400023622	4	8358204 - 8359541	438	2	145	16.1	6.92
StHsp20-12	PGSC0003DMG400024099	4	60045801 - 60046965	882	2	293	33.1	6.00
StHsp20-13	PGSC0003DMG400031133	4	61728105 - 61729376	510	2	169	18.6	5.30
StHsp20-14	PGSC0003DMG400009996	4	71887737 - 71891840	462	2	153	16.7	7.71
StHsp20-15	PGSC0003DMG400010001	4	71971113 - 71980699	912	13	303	34.0	9.68
StHsp20-16	PGSC0003DMG400011977	5	11646812 - 11649618	681	3	226	26.2	9.39
StHsp20-17	PGSC0003DMG400030427	6	56896895 - 56897612	465	1	154	17.7	5.57
StHsp20-18	PGSC0003DMG400030426	6	56893292 - 56894077	465	1	154	17.6	5.83
StHsp20-19	PGSC0003DMG400030339	6	56900911 - 56901677	465	1	154	17.7	6.20
StHsp20-20	PGSC0003DMG400030340	6	56905147 - 56905872	465	1	154	17.6	7.91
StHsp20-21	PGSC0003DMG400030341	6	56907909 - 56908742	465	1	154	17.6	5.57
StHsp20-22	PGSC0003DMG401017288	7	50794176 - 50799268	753	6	250	27.9	9.19
StHsp20-23	PGSC0003DMG400019265	7	54203551 - 54205245	573	2	190	21.8	5.49
StHsp20-24	PGSC0003DMG400021737	8	34366816 - 34367720	477	1	158	17.7	6.17
StHsp20-25	PGSC0003DMG400008187	8	34544813 - 34545655	477	1	158	17.6	6.17
StHsp20-26	PGSC0003DMG400004808	8	52375001 - 52376265	636	2	211	23.9	6.45
StHsp20-27	PGSC0003DMG400004807	8	52380425 - 52381599	588	2	195	21.4	8.65
StHsp20-28	PGSC0003DMG400004806	8	52390914 - 52392554	513	2	170	18.5	5.05
StHsp20-29	PGSC0003DMG400020341	9	829873 - 831558	486	2	161	19.2	9.77
StHsp20-30	PGSC0003DMG400011719	9	888985 - 890624	582	2	193	22.7	7.06
StHsp20-31	PGSC0003DMG400002009	9	6945529 - 6946992	744	2	247	27.5	5.53
StHsp20-32	PGSC0003DMG400011628	9	11636814 - 11637710	465	1	154	17.5	6.21
StHsp20-33	PGSC0003DMG400011630	9	11675983 - 11676827	495	1	164	18.8	6.15
StHsp20-34	PGSC0003DMG400011631	9	11678942 - 11679778	474	1	157	17.9	6.21
StHsp20-35	PGSC0003DMG400011632	9	11684152 - 11685032	465	1	154	17.5	6.21
StHsp20-36	PGSC0003DMG400014956	9	31436185 - 31436781	402	1	133	15.3	6.19
StHsp20-37	PGSC0003DMG400017098	9	31745004 - 31745667	423	1	140	16.2	5.63
StHsp20-38	PGSC0003DMG400019136	10	50699792 - 50700705	705	1	234	27.3	9.56
StHsp20-39	PGSC0003DMG400019137	10	50704357 - 50705144	588	1	195	22.5	9.88
StHsp20-40	PGSC0003DMG400007210	10	59676867 - 59677589	444	2	147	16.5	8.89
StHsp20-41	PGSC0003DMG400009255	11	13518704 - 13519520	594	1	197	22.4	5.41
StHsp20-42	PGSC0003DMG400018717	11	42985862 - 42987493	744	2	247	27.5	8.32
StHsp20-43	PGSC0003DMG400002928	12	3139670 - 3140616	468	1	155	17.7	5.27
StHsp20-44	PGSC0003DMG400039484	12	3142147 - 3142608	462	1	153	17.3	5.54
StHsp20-45	PGSC0003DMG400047019	12	19504973 - 19513520	909	9	302	33.8	8.24
StHsp20-46	PGSC0003DMG400028624	12	49808899 - 49810200	690	2	229	26.2	8.44
StHsp20-47	PGSC0003DMG402010796	12	54506438 - 54507366	750	2	249	28.2	8.23
StHsp20-48	PGSC0003DMG400029311	12	57570831 - 57572324	423	6	140	15.5	6.19

### *StHsp20* gene structure

**Fig. 1:**
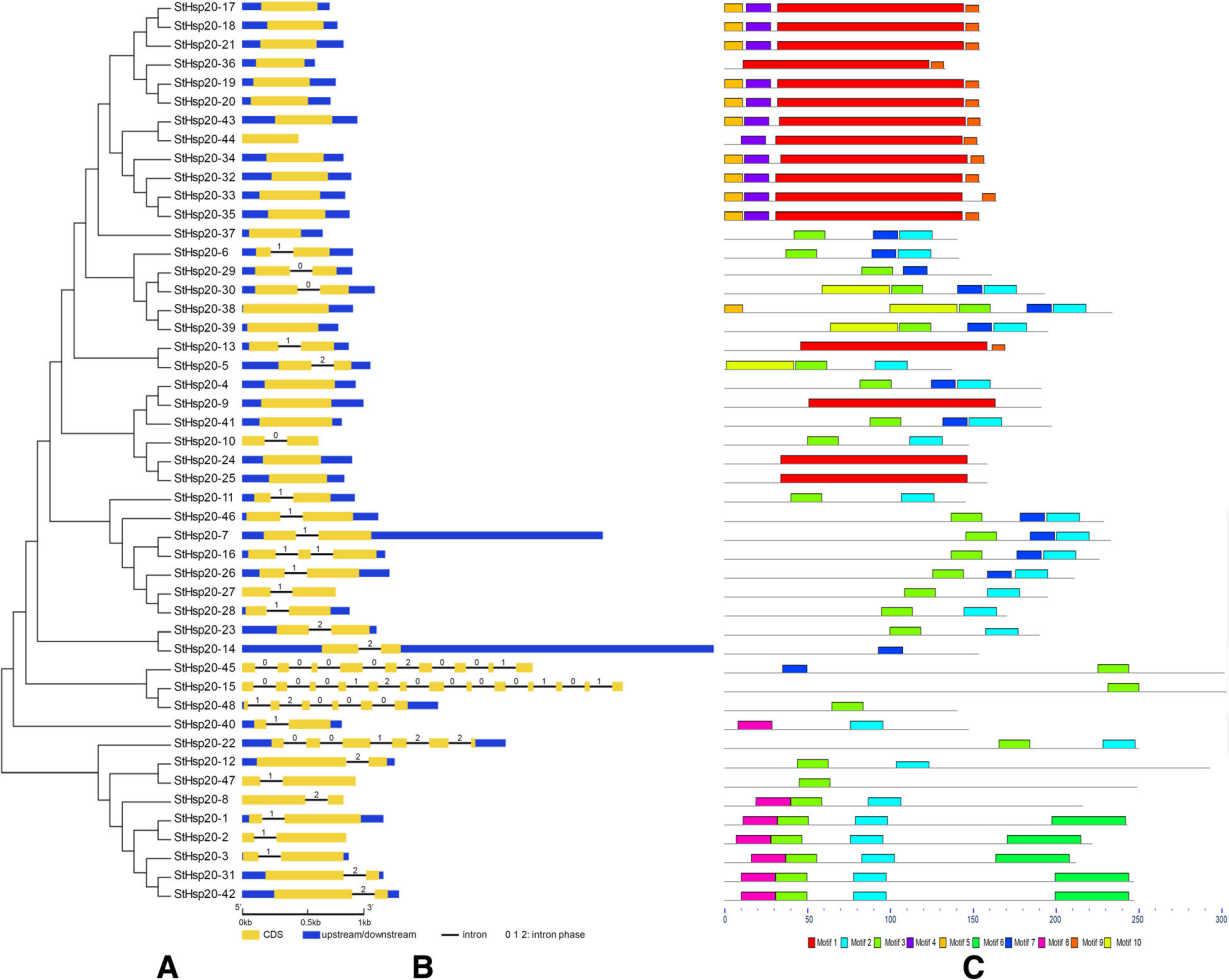
Phylogenetic relationship, gene structure and conserved motif analysis of *StHsp20* genes. **a** Phylogenetic tree of 48 StHsp20 proteins. The unrooted neighbor-joining phylogenetic tree was constructed with MEGA6 using full-length amino acid sequences of 48 StHsp20 proteins, and the bootstrap test replicate was set as 1000 times. **b** Exon/intron organization of *StHsp20* genes. Yellow boxes represent exons and black lines with same length represent introns. The upstream/downstream region of *StHsp20* genes are indicated in blue boxes. The numbers of 0, 1, and 2 represent the splicing phase of intron. The length of exons can be inferred by the scale at the bottom. **c** Distributions of conserved motifs in *StHsp20* genes. Ten putative motifs are indicated in different colored boxes. For details of motifs refer to Table 2

Structures and phases of introns/exons were determined by the alignment of genomic DNA with full-length cDNA of *StHsp20s*. Among the *StHsp20* genes, nearly half (20, 41.7%) were intronless, 23 (47.9%) had one intron, and only 5 genes (10.4%), *StHsp20-15* (12 introns), *StHsp20-22* (5 introns), *StHsp20-15* (12 introns), *StHsp20-45* (8 introns) and *StHsp20-48* (5 introns), had two or more introns (Fig. 1). Interestingly, all of the tandem duplicated genes were intronless and the pair of segmentally duplicated genes, *StHsp15* and *StHsp48*, had multiple introns. *StHsp48* was shorter than *StHsp15* in sequence length, but shared a highly conserved region with *StHsp15.* The conserved region possessed the same intron phase (1, 2, 0, 0 and 0). The result suggested a particular phylogenetic relationship between the two segmentally duplicated genes.

**Fig. 2:**
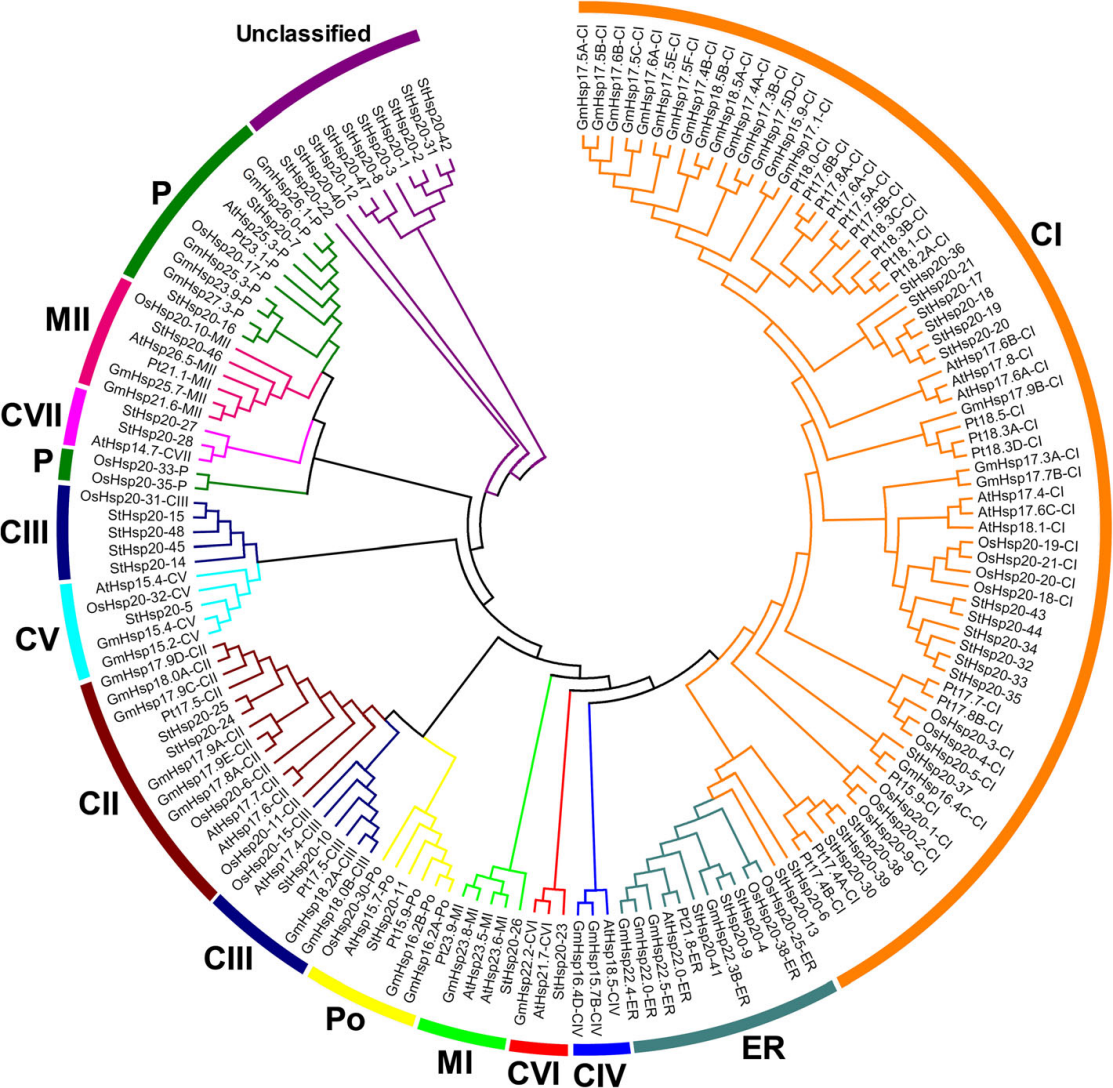
Phylogenetic tree of Hsp20s from *Arabidopsis*, *Populus*, soybean, rice and potato The phylogenetic tree was constructed using the NJ (Neighbor-joining) method with 1000 bootstrap replications. The 12 subfamilies were distinguished in different colors, and the unclassified StHsp20s were colored in purple

The conserved motifs of StHsp20 proteins were identified by MEME website, and 10 were found. The lengths of these conserved motifs varied from 8 to 113 amino acids. Details of the 10 putative motifs are outlined in Table 2. Based on analyses of Pfam, CDD and SMART, Motif 1 completely corresponded to the region of the conserved ACD. The full sequences of Motifs 2, 3 and 7 together formed a highly conserved complete ACD. The majority of the StHsp20 proteins (58.3%) contained Motif 1 or the combination of Motifs 2, 3 and 7. Other StHsp20 proteins lacked the complete combination of motifs. StHsp20-1, 2, 3, 8, 31, 40 and 42 contained Motif 8, which was predicted to be a transmembrane region. Ten StHsp20 proteins could not be classified with other types of StHsp20 proteins (Fig. 2). The different compositions of the ACD domain may indicate functional diversity. The same group of StHsp20 proteins in the phylogenetic tree shared common motifs and indicated they were highly conserved.

**Table 2:** List of the putative motifs of StHsp20 proteins

Motif	Width	Best possible match
1	113	FPPSSSRETSAFANTRIDWKETPEAHVFKVDVPGLKKEEVKVEVEEDRVLQISGERSREKEEKNDKWHRVERSSGKFMRRFRLPENAKMDQIKASMENGVLTVTVPKEEEKKP
2	20	DVDKIKAKMENGVLTVTVPK
3	19	ADLPGLKKEDVKVQVEDNG
4	15	SNIFDPFSLDVFDPF
5	11	MSLIPRFFGGR
6	45	TDDATIGTASVLAAKLKMPRKVMNMTLVALLVLGIGLVVSNKMKS
7	15	RSYGKFSTSFNLPEN
8	21	VYEDFVPSTELVQEEDSDTLL
9	8	VKSIDISG
10	41	PSHEFYLETPRSLIAPSLSFPHVPQYMAQIEYKETPEAHIF

### Phylogenetic analysis of *StHsp20* genes

To analyze the evolutionary relationships of *Hsp20* genes in potato, *Arabidopsis*, soybean, rice and *Populus*, an unrooted phylogenetic tree was constructed using full-length amino acid sequences. In total, 19 sequences from *Arabidopsis*, 22 sequences from rice, 47 sequences from potato, 46 sequences from soybean and 25 sequences from *Populus* were assessed in the phylogenetic tree (Fig. 2). The potato Hsp20 family member StHsp20-29 was excluded from the phylogenetic tree because it was too divergent to be aligned with other sequences. The 159 Hsp20s were classified into 12 distinct subfamilies, 71 cytosol Is (CIs), 13 CIIs, 11 CIIIs, 3 CIVs, 5 CVs, 3 CVIs, 3 CVIIs, 5 mitochondria Is (MIs), 6 MIIs, 12 plastids (Ps), 6 peroxisomes (Pos) and 11 endoplasmic reticulum (ERs). However, the remaining 10 potato Hsp20s could not be clustered into any subfamily. Except for the unclassified StHsp20s, 37 StHsp20s existed in 11 subfamilies, except for the CIV subfamily. Most of the Hsp20s, including 29 StHsp20s, were classified into CI–CVII, which indicated that cytosol might be the main functional area for plant Hsp20s. Remarkably, StHsp20 members were more closely related to those in the same subfamily from different species than to the other Hsp20s from the same species, which implied a relatively high synteny between the same Hsp20 subfamily across various species. It was interesting that the P and M (MI and MII) subfamily members had a close relationship with each other, which indicated that the M subfamily evolved from the P subfamily once again [6]. No Hsp20 protein of monocotyledon (rice) was found in CIV subfamily. According to previous study [35], CIV subfamily of Hsp20s existed only in dicotyledon.

A close relationship between the phylogenetic classification and intron pattern existed. According to previous research, three patterns were proposed. Pattern 1 means no intron, Pattern 2 means one intron, and Pattern 3 means more than one intron [24]. Most *StHsp20* members of the CI subfamily lacked introns, and the CII and ER subfamilies had no introns. However, all of the members of the CV, CVI, CVII, Po, MI and MII subfamilies had one intron, which indicated a close phylogenetic relationship (Fig. 1; Table 1). In addition, three genes (*StHsp15*, *StHsp45* and *StHsp48*) belonging to the CIII subfamily had 12, 8 and 5 introns, respectively (Fig. 1; Table 1). The presence of multiple introns indicated a particular phylogenetic status.

### Chromosomal location and gene duplication of *StHsp20s*

**Fig. 3:**
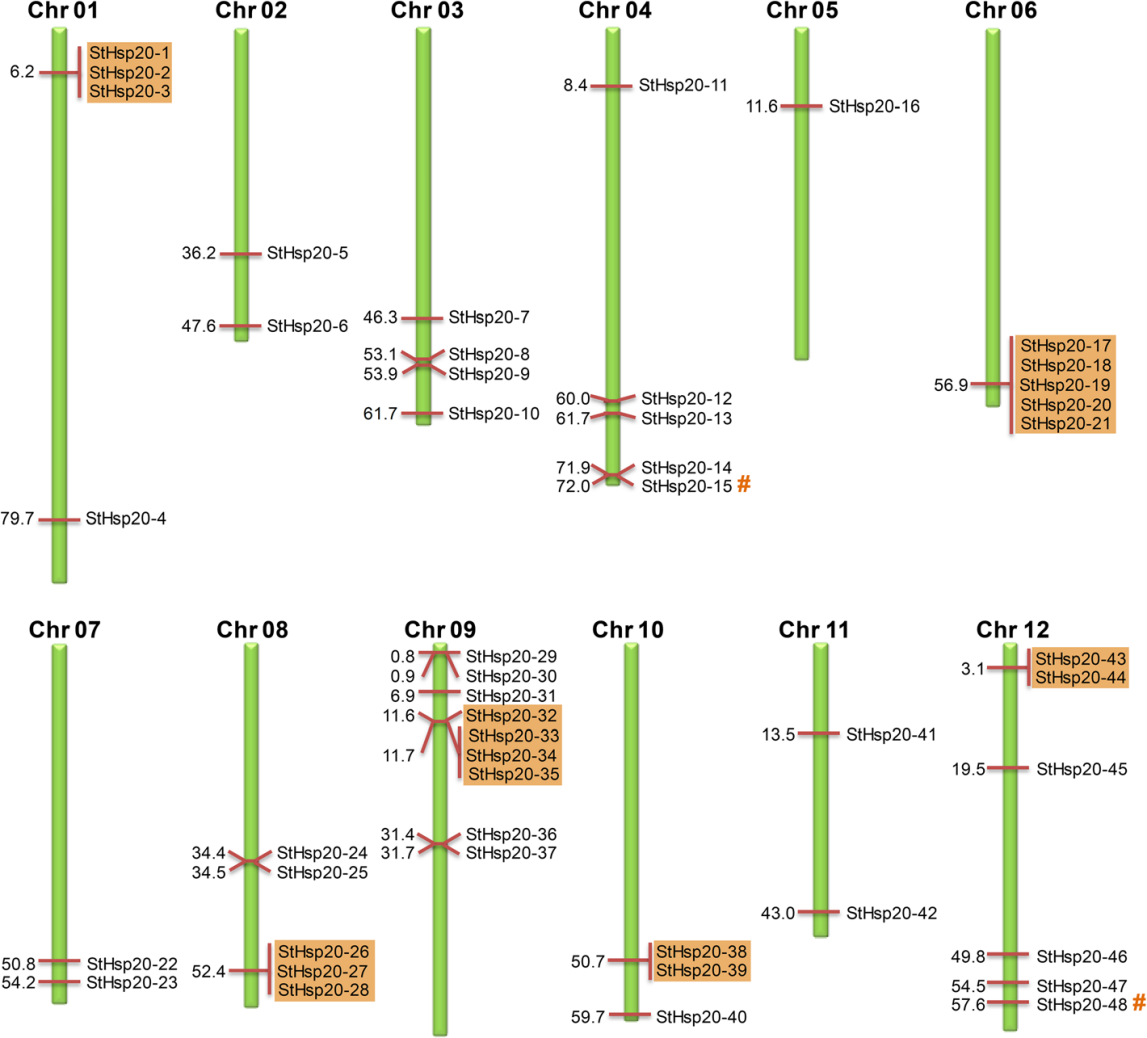
Chromosomal location and gene duplication of *StHsp20s*. The tandem duplicated genes are marked by orange rectangles and segmentally duplicated genes are indicated by symbol #

The 48 *StHsp20* genes were distributed on 12 potato chromosomes randomly (Fig. 3). The majority of *StHsp20* genes were located on the proximate or the distal ends of the chromosomes. The maximum number of nine predicted *StHsp20* genes, scattered in two clusters, were present on chromosome 9, and only one gene existed on chromosome 5.

During the progress of evolution, both tandem duplication and segmental duplication contribute to the generation of gene family [42]. Thus, we analyzed the duplication events of *StHsp20* genes. Based on the defined criteria, 19 genes (39.6%) were confirmed to be tandem duplicated genes. Two separate pairs of tandem duplicated genes located on chromosome 10 and chromosome 12. Two groups of three tandem duplicated genes located on chromosome 1 and 8. Five and four tandem duplicated genes located on chromosome 6 and 9, separately. Additionally, two genes (*StHsp20-15* and *StHsp20-48*) were segmentally duplicated genes, and the length of segmentally duplicated chromosome was 625 kb. Segmental duplication only accounted for 4.2% of the *StHsp20* genes. Based on above results, it could be inferred that tandem duplication and segmental duplication contribute to the expansion of *StHsp20* family together, but the former played a predominant role.

### Stress-related *cis*-elements in *StHsp20* promoters

**Fig. 4:**
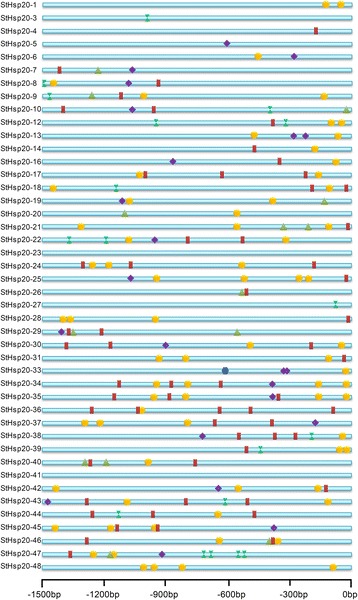
Predicted *cis*-elements in *StHsp20* promoters. Promoter sequences (−1500 bp) of 44 *StHsp20* genes (promoter regions of *StHsp20-2*, *StHsp20-11*, *StHsp20-15* and *StHsp20-32* were absent) are analyzed by PlantCARE. The upstream length to the translation start site can be inferred according to the scale at the bottom

To further study the potential regulatory mechanisms of *StHsp20* during abiotic stress responses, the 1.5-kb upstream sequences from the translation start sites of *StHsp20* genes (promoter regions of *StHsp20-2*, *StHsp20-11*, *StHsp20-15* and *StHsp20-32* were absent) were submitted into PlantCARE to detect the *cis*-elements. Six abiotic stress response elements, ABA-responsive elements, DRE, HSE, LTRE, TC-rich repeat and W-box, were analyzed and displayed in Fig. 4. Except for StHsp20-23 and StHsp20-41, the other StHsp20s possessed at least 1 stress-response-related *cis*-element, which indicated that the expressions of *StHsp20*s were associated with these abiotic stresses. In total, 32 *StHsp20*s (72.8%) had one or more HSEs, suggesting a potential heat-stress response under high temperature conditions. One to two LTREs existed in 11 *StHsp20s*, and 1 DRE was found in *StHsp20-33*. TC-rich repeats and W-boxes were located in 34 and 13 *StHsp20*s, respectively. Anyhow, the *cis*-element analysis illustrated that *StHsp20* genes could respond to different abiotic stresses.

### Expression patterns of *StHsp20* genes in different tissues

**Fig. 5:**
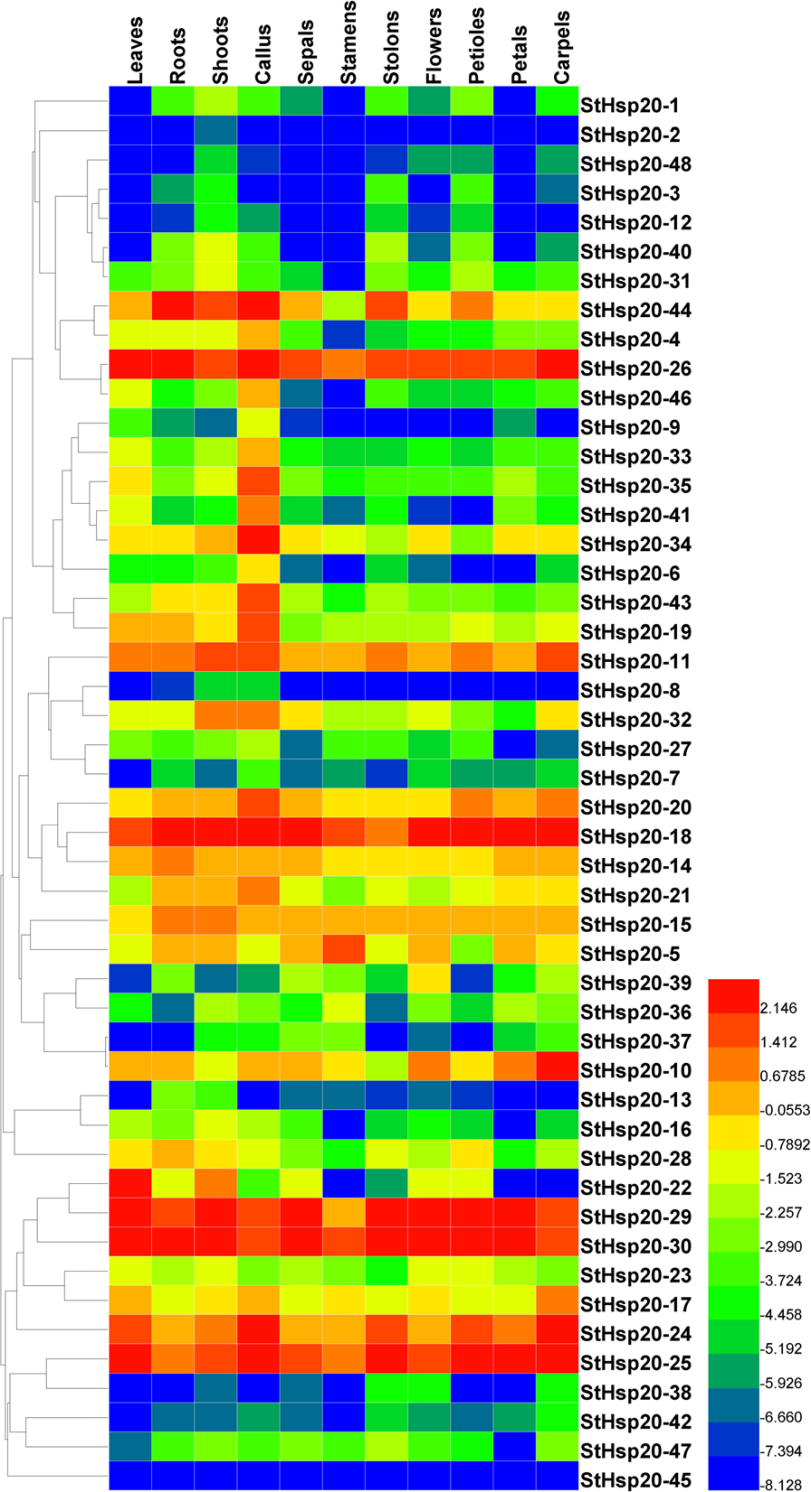
Expression profiles of *StHsp20s* in different tissues and organs. FPKM values of *StHsp20* genes were transformed by log2 and the heatmap was constructed by HemI software

Using the RNA-seq data, a heatmap of 48 *StHsp20* genes, represented by FPKM values in different tissues and organs, was established by HemI (Fig. 5). Most of *StHsp20s* were expressed in one tissue at least, except for *StHsp20-2* and *StHsp20-45*, which were barely expressed in any tissue or organ. Six genes including *StHsp20-18*, *StHsp20-24*, *StHsp20-25*, *StHsp20-26*, *StHsp20-29* and *StHsp20-30*, were highly expressed in all of the tissues. Some *StHsp20* genes showed similar expression patterns in various tissues. *StHsp20-1*, *StHsp20-3*, *StHsp20-12*, *StHsp20-40* and *StHsp20-48* showed relatively high expression levels in vegetative organs, such as shoots, stolons and petioles, but undetectable levels in leaves, sepals, stamens, flowers and petals. *StHsp20-6*, *StHsp20-9*, *StHsp20-19*, *StHsp20-33*, *StHsp20-34*, *StHsp20-35*, *StHsp20-41* and *StHsp20-43* were highly expressed in callus. *StHsp20-7*, *StHsp20-8*, *StHsp20-11*, *StHsp20-27* and *StHsp20-32* exhibited high expression levels in shoots and callus.

### Expression profiles of *StHsp20s* under abiotic stress

**Fig. 6:**
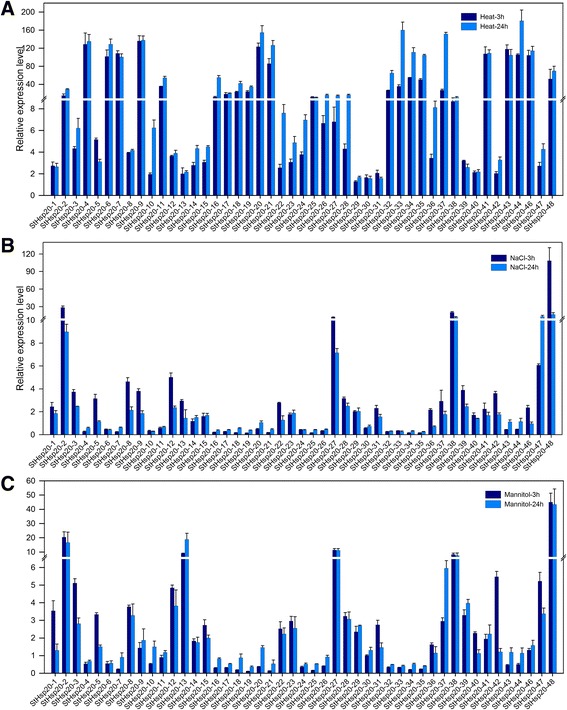
Expression profiles of *StHsp20* genes under heat, salt and drought stresses. Quantitative RT-PCR was used to investigate the expression levels of each *StHsp20* gene. To calculate the relative expression level, the expression of each gene under control treatment was set as 1. The results were represented by mean ± standard deviation. The reference gene used in qRT-PCR was *ef1α*

To further explore the expression changes in the *StHsp20* genes under various abiotic stresses including heat, salt and drought, qRT-PCR was used to investigate the transcript levels of each *StHsp20* gene with 3 biological repetitions and 2 technical repetitions. Generally, the relative expression level of the *StHsp20* genes under all stress conditions fluctuated during the 24-h treatments (Fig. 6). The relative expression level of *StHsp20-45* was not shown because the non-specific primers may lead to unreliable results. Most of the *StHsp20* genes were sensitive to heat stress, and none of the genes were down-regulated, but *StHsp20-29* and *StHsp20-30* showed no differences after being treated for 3 h and 24 h under heat stress. The expression levels of *StHsp20-10* and *StHsp20-13* were up-regulated only after a 24-h heat treatment. The relative expression levels of 14 *StHsp20* genes (*StHsp20-4*, *6*, *7*, *9*, *20*, *21*, *33*, *34*, *35*, *37*, *41*, *43*, *44* and *46*) were extremely up-regulated (more than 100-fold) under heat stress compared with the control.

Although the *Hsp20* family is generally induced by heat stress, we also determined whether the family is involved in responses to salt and drought stresses. The expression levels of *StHsp20* genes under salt and drought stresses varied among the 47 members. The expression pattern of each *StHsp20* was different from that under heat stress. Nearly half of the *StHsp20* (40.4%) genes were down-regulated after being treated for 3 h or 24 h. Six genes (*StHsp20-11*, *14*, *15*, *23*, *30* and *40*) and 10 genes (*StHsp20-4*, *6*, *9*, *10*, *11*, *14*, *30*, *36*, *44* and *46*) were not sensitive to salt and drought stresses, respectively. The remaining *StHsp20s* were up-regulated under salt and drought stresses, but the changes were not as extreme as that under heat stress. The differential expression patterns compared with those under heat stress indicated there were different response and regulatory mechanisms of the StHsp20 family under various abiotic stress conditions.

RNA-seq data of *StHsp20* under abiotic stress after treated for 24 h was collected from PGSC and processed to compare the expression abundance with that of qRT-PCR. The relative expression level was represented by stress/control (Additional file 4: Figure S1). However, two sections of the results were not completely in accordance with each other. Under heat stress, only 10 of the *StHsp20* genes showed high expression level while 20 *StHsp20s* showed low expression level. High expression levels were confirmed in nearly half of *StHsp20s* under salt and drought stresses, meanwhile 3 and 2 *StHsp20s* exhibited low expression level respectively. In a word, compared the two set of results from RNA-seq and qRT-PCR, 4 genes (*StHsp20-5, 10, 13 and  22*) showed a similar expression pattern under 3 abiotic stresses, and 5 genes (*StHsp20-7, 15, 29, 42 and 47*) had similar expression pattern under salt and drought stress.

## Discussion

Hsp20s, as molecular chaperone, inhibit the irreversible aggregation of denaturing proteins, thus enhance the thermotolerance of plant [16]. With the availabilities of the whole genome sequence of many plants, several Hsp20 families have been identified, such as *Arabidopsis*, rice, *Populus*, pepper and tomato [23, 26, 27, 36]. However, little is known about Hsp20 family in potato.

The current study identified 48 *StHsp20* genes, and analyzed their structure, chromosomal location, phylogeny, gene duplication, stress-related *cis*-elements and expression patterns in different tissues and abiotic stresses. The study provides comprehensive information on the *StHsp20* gene family and will aid in understanding the functional divergence of *Hsp20* genes in potato.

Previous research identified 19, 39, 35 and 42 *Hsp20* genes in *Arabidopsis*, rice, pepper and tomato, respectively [24, 26, 27, 35]. The low number of *Hsp20* genes in *Arabidopsis* is related to its small genome. Forty-eight *Hsp20* genes were identified in potato, which was close to the numbers found in pepper and tomato, which also belong to *Solanaceae*.

Gene organization plays a vital role in the evolution of multiple gene families [43]. In this study the percentage of intronless *StHsp20* genes is similar to that of pepper (45.71%) [26] and tomato (30.95%) [27]. Additionally, *StHsp20* genes of the CII and ER subfamilies, as well as most *StHsp20* genes of the CI subfamily, were intronless (Fig. 2, Table 1). Members of the CV, CVI, MI, MII, P and Po subfamilies had only one intron. The results are also in accordance with that in pepper and tomato. Additionally, similar motif arrangements were found in the same subfamily members (Figs. 1c, 2). This correlation between intron numbers and motif arrangement further confirmed the classifications of the *StHsp20* genes. In some studies, genes with few or no introns were considered to have enhanced expression levels in plants [44, 45]. To response to various stresses timely, genes must be rapidly activated, which would be assisted by a compact gene structure with less introns [46]. Most of the *StHsp20* genes were highly induced under heat stress (Fig. 6), which may approve the above standpoints in other research.

In earlier studies, *Arabidopsis Hsp20* genes were classified into seven subfamilies (CI, CII, CIII, M, P, ER and Po), and five genes could not be clustered into any subfamily [23]. Subsequently, four new nucleocytoplasmic subfamilies (CIV, CV, CVI and CVII) and a mitochondrial subfamily (MII) were identified [35]. In our study, the phylogenetic tree showed that *Hsp20* genes were classified into 12 distinct subfamilies. The *StHsp20* genes existed in 11 of the 12 subfamilies. There was no *Hsp20* gene of potato in the CIV subfamily, which may be the result of gene loss during evolution.

Most of the *StHsp20* genes (61.7%) were grouped into a nucleocytoplasmic subfamily, which was also illustrated in *Arabidopsis*, pepper and tomato [23, 26, 27]. Among these subfamilies, CI was the largest subfamily, containing 18 *StHsp20* genes. Based on these results, we inferred that, because proteins are mainly synthesized in the cytoplasm, this could be the primary place for Hsp20 proteins to interact with denatured proteins, preventing inappropriate aggregation and degradation. Furthermore, the *Hsp20* genes in the same subfamily from different species were more similar than those of the same species but belonging to various subfamilies. The finding indicated that synteny might exist in *Arabidopsis*, *Populus*, rice and soybean Hsp20 proteins, and that Hsp20 subfamilies diversified before the divergence within these species.

The expansions of gene families and genome evolutionary mechanisms mainly depend on gene duplication events [47]. The major duplication patterns are tandem duplication and segmental duplication [48]. In this research, 48 *StHsp20* genes were located unevenly on 12 potato chromosomes, and most of the *StHsp20* genes were located on the terminal regions of the chromosomes. Although the genome size of potato is almost 7 times that of *Arabidopsis*, the number of *Hsp20* genes in potato (48 genes) is only 2.5 times that in *Arabidopsis* (19 genes). This could be the result of different whole genome duplication events in *Arabidopsis* and potato. A total of 21 *StHsp20* duplicated genes were detected in potato, including one pair of segmentally duplicated genes (*StHsp20-15* and *StHsp20-48*) and four tandem duplicated gene groups (Fig. 3), which revealed that both tandem and segmental duplications contributed to the evolution of *Hsp20* genes in potato. Similar expression patterns under various abiotic stresses were found within the tandem duplicated gene groups (Fig. 6). The similar expression patterns indicated the analogous functions and structures of tandem duplicated *StHsp20* genes. The redundancies of functions and similarities of structures may reflect shared induction mechanisms.

The expression patterns of *Hsp20* genes in different tissues have been described in many species, such as *Arabidopsis*, rice, pepper and tomato [24, 26, 27, 35]. There is no uniform gene expression pattern for plant *Hsp20* genes. According to the RNA-seq data of potato, several *StHsp20* genes such as *StHsp20-22* and *StHsp20-41*, exhibited incongruous expression patterns in various tissues, indicating that different StHsp20 proteins may have diverse functions. Three genes, *StHsp20-18, StHsp20-26* and *StHsp20-30*, were highly and indiscriminately expressed in all of the investigated tissues under normal condition. Similar with several *Hsp20* genes in soybean, the three *StHsp20s* showed specific housekeeping expression activity [25].

qRT-PCR was used to investigate the transcript levels of each *StHsp20* under different abiotic stresses. The two genes (*StHsp20-29* and *StHsp20-30*) with distinctive expression patterns were highly expressed in all of the investigated tissues, but no induction was observed under heat stress. Thus, we may assume that the two genes are lacking of chaperone activities. The results confirmed the association of potato Hsp20 proteins with thermotolerance; however, the existence of numerous Hsp20s may lead to functional redundancy [6]. In addition, similar expression patterns in *StHsp20* genes may be caused by shared induction mechanisms. Because the heat shock response network involves heat shock proteins and heat shock transcription factors (Hsfs), the expression levels of *Hsp20* genes rely heavily on the activation of Hsfs under heat stress. During a 24-h heat treatment, the *StHsp20* genes showed different transcript accumulation levels. It was reported that the same set of *Hsps* could be regulated by different Hsfs on transcription level [49, 50], which indicated that *StHsp20* genes are specifically controlled by various Hsfs. The differences in transcription levels of *StHsp20s* may be the reflection of different upstream regulating genes of *Hsfs*.

Based on qRT-PCR, all of the *StHsp20* genes responded to salt and drought stress; however, the expression level of several *StHsp20s* was down-regulated (Fig. 6). Under heat stress, Hsfs are activated and bound to HSEs in the *Hsp20* gene promoters to regulate the expressions of downstream genes. Nevertheless, various *cis*-elements were found in promoter regions of *StHsp20s* (Fig. 4), and these are involved in the responses of *StHsp20* genes to other abiotic stresses. Thus, *StHsp20* genes could be induced by both heat stress and other abiotic stresses. The multiple abiotic stress responses of *StHsp20* genes reflected an interconnected induction mechanism involving *Hsf* transcription factors.

Compared with expression pattern represented by RNA-seq data, the expression profile generated by qRT-PCR was not completely equal to that. The difference of expression pattern may be caused by multiple reasons. Although the same plant material (DM) was used for research, only aboveground part of plant was collected in our research, while the whole plant was sampled for RNA sequencing. Specific to heat stress, the plant was treated for 24-h in normal photoperiod of 16 h light/8 h dark in our study, but the plant for RNA sequencing was treated in the dark. The potato RNA-seq data used in our research was presented as FPKM. Compared with raw read counts, FPKM value can better reduce sample differences. However, the FPKM value could be significantly changed due to highly expressed genes [51]. The bias of FPKM value leads to different expression compared with qRT-PCR.

## Conclusions

Here, a genome-wide analysis of potato *Hsp20* family was performed, and 48 *StHsp20* genes were confirmed. Subsequently, analyses of *StHsp20* genes on gene structures, phylogeny, chromosomal location, gene duplication, stress-related *cis*-elements, expression patterns in different tissues and abiotic stresses, were conducted based on bioinformatics and qRT-PCR methods. Most of *StHsp20* genes were sensitive to heat stress and were up-regulated rapidly, indicating that *StHsp20* genes play important roles in the acquired thermotolerance of potato. The study provides comprehensive information on the *StHsp20* gene family in potato and will aid in determining the *StHsp20* gene functions.

## Additional files

Additional file 1: Table S1. The IDs of *Hsp20* genes from *Arabidopsis*, soybean, rice and *Populus*. (DOCX 28 kb)

Additional file 2: Table S2. FPKM values of 48 *StHsp20* genes in various potato tissues. (XLSX 15 kb)

Additional file 3: Table S3. The primer sequences of 48 *StHsp20* genes used for qRT-PCR. (DOCX 15 kb)

Additional file 4: Figure S1. Heatmap of *StHsp20s* under heat, salt and drought stress. (TIFF 327 kb)

## Abbreviations

AA: Amino acid
ABRE: ABA responsive element
ACD: Alpha-crystallin domain
BLASTP: Basic local alignment search tool-protein
CRI: Conserved region I
CRII: Conserved region II
DRE: Dehydration-responsive element
FPKM: Fragments per kilobase of transcript per million mapped reads
GSDS: Gene structure display server
HMM: Hidden markov model
HSE: Heat stress element
Hsfs: Heat shock transcription factors
Hsps: Heat shock proteins
LTRE: Low temperature responsive element
MW: Molecular weight
ORF: Open reading frame
PGDD: Plant genome duplication database
PGSC: Potato genome sequencing consortium
PI: Isoelectric point
qRT-PCR: Quantitative real-time polymerase chain reaction
sHsp: Small heat shock proteins

## References

[1] Giorno F, Wolters-Arts M, Grillo S, Scharf KD, Vriezen WH, Mariani C (2009). Developmental and heat stress-regulated expression of HSFa2 and small heat shock proteins in tomato anthers. J Exp Bot.

[2] Wang W, Vinocur B, Altman A (2003). Plant responses to drought, salinity and extreme temperatures: towards genetic engineering for stress tolerance. Planta.

[3] Mittler R (2006). Abiotic stress, the field environment and stress combination. Trends Plant Sci.

[4] Herman DJ, Knowles LO, Knowles NR (2017). Heat stress affects carbohydrate metabolism during cold-induced sweetening of potato (*Solanum tuberosum* L.). Planta.

[5] De MA (1999). Heat shock proteins: facts, thoughts, and dreams. Shock.

[6] Waters ER (2013). The evolution, function, structure, and expression of the plant sHsps. J Exp Bot.

[7] Mogk A, Bukau B (2017). Role of sHsps in organizing cytosolic protein aggregation and disaggregation. Cell Stress Chaperon..

[8] Wang W, Vinocur B, Shoseyov O, Altman A (2004). Role of plant heat-shock proteins and molecular chaperones in the abiotic stress response. Trends Plant Sci.

[9] Sarkar NK, Kim YK, Grover A (2009). Rice sHsp genes: genomic organization and expression profiling under stress and development. BMC Genomics.

[10] Waters ER, Lee GJ, Vierling E (1996). Evolution, structure and function of the small heat shock proteins in plants. J Exp Bot.

[11] Sung DY, Kaplan F, Lee KJ, Guy CL (2003). Acquired tolerance to temperature extremes. Trends Plant Sci.

[12] Vierling E (2003). The roles of heat shock proteins in plants. Annu Rev Plant Biol.

[13] Charng YY, Liu HC, Liu NY, Hsu FC, Ko SS (2006). (2006). Arabidopsis Hsa32, a novel heat shock protein, is essential for acquired thermotolerance during long recovery after acclimation. Plant Physiol.

[14] Lee GJ, Vierling E (2000). A small heat shock protein cooperates with heat shock protein 70 systems to reactivate a heat-denatured protein. Plant Physiol.

[15] Cashikar AG, Duennwald M, Lindquist SL (2005). A chaperone pathway in protein disaggregation. Hsp26 alters the nature of protein aggregates to facilitate reactivation by Hsp104. J Biol Chem.

[16] Haslbeck M, Vierling E (2015). A first line of stress defense: small heat shock proteins and their function in protein homeostasis. J Mol Biol.

[17] Kirschner M, Winkelhaus S, Thierfelder JM, Nover L (2000). Transient expression and heat-stress-induced co-aggregation of endogenous and heterologous small heat-stress proteins in tobacco protoplasts. Plant J.

[18] Giese KC, Vierling E (2004). Mutants in a small heat shock protein that affect the oligomeric state. Analysis and allele-specific suppression. J Biol Chem.

[19] Basha E, Friedrich KL, Vierling E (2006). The N-terminal arm of small heat shock proteins is important for both chaperone activity and substrate specificity. J Biol Chem.

[20] Jaya N, Garcia V, Vierling E, Lorimer GH (2009). Substrate binding site flexibility of the small heat shock protein molecular chaperones. Proc Natl Acad Sci U S A.

[21] Bondino HG, Valle EM, Have AT (2012). Evolution and functional diversification of the small heat shock protein/α-crystallin family in higher plants. Planta.

[22] Basha E, O’Neill H, Vierling E (2012). Trends Biochem Sci.

[23] Scharf KD, Siddique M, Vierling E (2001). The expanding family of *Arabidopsis thaliana* small heat stress proteins and a new family of proteins containing alpha-crystallin domains (acd proteins). Cell Stress Chaperon..

[24] Ouyang Y, Chen J, Xie W, Wang L, Zhang Q (2009). Comprehensive sequence and expression profile analysis of hsp20 gene family in rice. Plant Mol Biol.

[25] Lopes-Caitar VS, Carvalho MCD, Darben LM, Kuwahara MK, Nepomuceno AL, Dias WP, Abdelnoor RV, Marcelino-Guimarães FC (2013). Genome-wide analysis of the Hsp20 gene family in soybean: comprehensive sequence, genomic organization and expression profile analysis under abiotic and biotic stresses. BMC Genomics.

[26] Guo M, Liu JH, Lu JP, Zhai YF, Wang H, Gong ZH, Wang SB, Lu MH (2015). Genome-wide analysis of the CaHsp20 gene family in pepper: comprehensive sequence and expression profile analysis under heat stress. Front Plant Sci.

[27] Yu J, Cheng Y, Feng K, Ruan M, Ye Q, Wang R, Li Z, Zhou G, Yao Z, Yang Y, Wan H (2016). Genome-wide identification and expression profiling of tomato Hsp20 gene family in response to biotic and abiotic stresses. Front Plant Sci.

[28] Momčilović I, Pantelić D, Zdravković-Korać S, Oljača J, Rudić J, Fu J (2016). (2016). Heat-induced accumulation of protein synthesis elongation factor 1a implies an important role in heat tolerance in potato. Planta.

[29] Childs KL, Cepela J, Crisovan E (2014). Spud db: a resource for mining sequences, genotypes, and phenotypes to accelerate potato breeding. Plant Genome.

[30] Hu B, Jin J, Guo AY, He Z, Luo J, Gao G (2015). GSDS 2.0: an upgraded gene feature visualization server. Bioinformatics.

[31] Voorrips RE (2002). Mapchart: software for the graphical presentation of linkage maps and qtls. J Hered.

[32] Gu Z, Cavalcanti A, Chen FC, Bouman P, Li WH (2002). Extent of gene duplication in the genomes of drosophila, nematode, and yeast. Mol Biol Evol.

[33] Yang S, Zhang X, Yue JX, Tian D, Chen JQ (2008). Recent duplications dominate nbs-encoding gene expansion in two woody species. Mol Gen Genomics.

[34] Wang L, Guo K, Li Y, Tu Y, Hu H, Wang B, Cui X, Peng L (2010). Expression profiling and integrative analysis of the cesa/csl superfamily in rice. BMC Plant Biol.

[35] Siddique M, Gernhard S, Koskulldöring PV, Vierling E, Scharf KD (2008). The plant sHsp superfamily: five new members in *Arabidopsis thaliana* with unexpected properties. Cell Stress Chaperon..

[36] Waters ER, Aevermann BD, Sanders-Reed Z (2008). Comparative analysis of the small heat shock proteins in three angiosperm genomes identifies new subfamilies and reveals diverse evolutionary patterns. Cell Stress Chaperon.

[37] Thompson JD, Gibson TJ, Plewniak F, Jeanmougin F, Higgins DG (1997). The clustal_x windows interface: flexible strategies for multiple sequence alignment aided by quality analysis tools. Nucleic Acids Res.

[38] Tamura K, Stecher G, Paterson D, Filipski A, Kumar S (2007). Mega6: molecular evolutionary genetics analysis software version 6.0. Mol Biol Evol.

[39] Lescot M, Déhais P, Thijs G, Marchal K, Moreau Y, Yves VDP, Pieree R, Stephane R (2002). Plantcare, a database of plant cis-acting regulatory elements and a portal to tools for in silico analysis of promoter sequences. Nucleic Acids Res.

[40] Murashige T, Skoog F (2006). A revised medium for rapid growth and bio assays with tobacco tissue cultures. Physiol Plant.

[41] Deng W, Wang Y, Liu Z, Cheng H, Xue Y (2013). Hemi: a toolkit for illustrating heatmaps. PLoS One.

[42] Cannon SB, Mitra A, Baumgarten A, Young ND, May G (2004). The roles of segmental and tandem gene duplication in the evolution of large gene families in *Arabidopsis thaliana*. BMC Plant Biol.

[43] Xu G, Guo C, Shan H, Kong H (2012). Divergence of duplicate genes in exon-intron structure. Proc Natl Acad Sci U S A.

[44] Ren X, Vorst O, Fiers M, Stiekema WJ, Nap J (2006). In plants, highly expressed genes are the least compact. Trends Genet.

[45] Chung BY, Simons C, Firth AE, Brown CM, Hellens RP (2006). Effect of 5’utr introns on gene expression in *Arabidopsis thaliana*. BMC Genomics.

[46] Jeffares DC, Penkett CJ, Bähler J (2008). (2008). Rapidly regulated genes are intron poor. Trends Genet.

[47] Vision TJ, Brown DG, Tanksley SD (2000). (2000). The origins of genomic duplications in *Arabidopsis*. Science.

[48] Kong H, Landherr LL, Frohlich MW, Leebensmack J, Ma H, Depamphilis CW (2007). Patterns of gene duplication in the plant skp1 gene family in angiosperms: evidence for multiple mechanisms of rapid gene birth. Plant J.

[49] Schramm F, Ganguli A, Kiehlmann E, Englich G, Walch D, Koskull-Döring PV (2006). The heat stress transcription factor Hsfa2 serves as a regulatory amplifier of a subset of genes in the heat stress response in *Arabidopsis*. Plant Mol Biol.

[50] Tang R, Zhu W, Song X, Lin X, Cai J, Wang M, Yang Q (2016). Genome-wide identification and function analyses of heat shock transcription factors in potato. Front Plant Sci.

[51] Kukurba KR, Montgomery SB (2015). RNA sequencing and analysis. Cold Spring Harb Protoc.

