# Supplementary material for: Genome-wide analysis of the potato Hsp20 gene family: identification, genomic organization and expression profiles in response to heat stress
Source: BMC Genomics. 2018 Jan 18;19:61. doi: 10.1186/s12864-018-4443-1 (PMC5774091; doi:10.1186/s12864-018-4443-1)
Supplement: Supplementary file 1 — The IDs of Hsp20 genes from Arabidopsis, soybean, rice and Populus. (DOCX 28 kb) [file 12864_2018_4443_MOESM1_ESM.docx]

| ***Arabidopsis*** | | | **Soybean** | | | **Rice** | | | ***Populus*** | |
| --- | --- | --- | --- | --- | --- | --- | --- | --- | --- | --- |
| **Gene name** | | **Gene ID** | **Gene name** | | **Gene ID** | **Gene name** | | **Gene ID** | **Gene name** | **Gene ID** |
| AtHsp17.4-CI | At3g46230 | | Glyma01g26570 | GmHsp16.4C-CI | | OsHsp20-1-CI | LOC_Os01g04340 | | Pt15.9-CI | 653054 |
| AtHsp17.6C-CI | At1g53540 | | Glyma13g27590 | GmHsp15.2-CV | | OsHsp20-2-CI | LOC_Os01g04350 | | Pt15.9-Po | 830535 |
| AtHsp18.1-CI | At5g59720 | | Glyma15g11360 | GmHsp15.4-CV | | OsHsp20-3-CI | LOC_Os01g04360 | | Pt17.4A-CI | 283987 |
| AtHsp17.6A-CI | At1g59860 | | Glyma12g06210 | GmHsp15.7B-CIV | | OsHsp20-4-CI | LOC_Os01g04370 | | Pt17.4B-CI | 762435 |
| AtHsp17.8-CI | At1g07400 | | Glyma04g38530 | GmHsp15.9-CI | | OsHsp20-5-CI | LOC_Os01g04380 | | Pt17.5A-CI | 738820 |
| AtHsp17.6B-CI | At2g29500 | | Glyma02g41150 | GmHsp16.2A-Po | | OsHsp20-6-CII | LOC_Os01g08860 | | Pt17.5B-CI | 574257 |
| AtHsp17.6-CII | At5g12020 | | Glyma14g39560 | GmHsp16.2B-Po | | OsHsp20-9-CI | LOC_Os02g03570 | | Pt17.5-CII | 832078 |
| AtHsp17.7-CII | At5g12030 | | Glyma11g14250 | GmHsp16.4D-CIV | | OsHsp20-10-MII | LOC_Os02g10710 | | Pt17.5-CIII | 712318 |
| AtHsp17.4-CIII | At1g54050 | | Glyma06g16490 | GmHsp17.1-CI | | OsHsp20-11-CII | LOC_Os02g12610 | | Pt17.6A-CI | 172186 |
| AtHsp15.4-CV | At4g21870 | | Glyma02g42000 | GmHsp17.3A-CI | | OsHsp20-15-CIII | LOC_Os02g54140 | | Pt17.6B-CI | 650093 |
| AtHsp21.7-CVI | At5g54660 | | Glyma08g07350 | GmHsp17.3B-CI | | OsHsp20-17-P | LOC_Os03g14180 | | Pt17.7-CI | 203787 |
| AtHsp14.7-CVII | At5g47600 | | Glyma08g07340 | GmHsp17.4A-CI | | OsHsp20-18-CI | LOC_Os03g15960 | | Pt17.8A-CI | 722968 |
| AtHsp23.5-MI | At5g51440 | | Glyma13g24510 | GmHsp17.4B-CI | | OsHsp20-19-CI | LOC_Os03g16020 | | Pt17.8B-CI | 549183 |
| AtHsp23.6-MI | At4g25200 | | Glyma07g32070 | GmHsp17.5A-CI | | OsHsp20-20-CI | LOC_Os03g16030 | | Pt18.0-CI | 723183 |
| AtHsp26.5-MII | At1g52560 | | Glyma07g32090 | GmHsp17.5B-CI | | OsHsp20-21-CI | LOC_Os03g16040 | | Pt18.1-CI | 721475 |
| AtHsp22.0-ER | At4g10250 | | Glyma07g32110 | GmHsp17.5C-CI | | OsHsp20-25-ER | LOC_Os04g36750 | | Pt18.2A-CI | 679803 |
| AtHsp15.7-Po | At5g37670 | | Glyma08g07330 | GmHsp17.5D-CI | | OsHsp20-30-Po | LOC_Os06g14240 | | Pt18.3A-CI | 579131 |
| AtHsp25.3-P | At4g27670 | | Glyma13g24440 | GmHsp17.5E-CI | | OsHsp20-31-CIII | LOC_Os06g41730 | | Pt18.3B-CI | 679801 |
| AtHsp18.5-CIV | At2g19310 | | Glyma13g24480 | GmHsp17.5F-CI | | OsHsp20-32-CV | LOC_Os07g33350 | | Pt18.3C-CI | 836664 |
|  |  | | Glyma07g32030 | GmHsp17.6A-CI | | OsHsp20-33-P | LOC_Os09g17660 | | Pt18.3D-CI | 563962 |
|  |  | | Glyma13g24460 | GmHsp17.6B-CI | | OsHsp20-35-P | LOC_Os10g07210 | | Pt18.5-CI | 659595 |
|  |  | | Glyma14g06900 | GmHsp17.7B-CI | | OsHsp20-38-ER | LOC_Os11g13980 | | Pt21.1-MII | 171902 |
|  |  | | Glyma06g05740 | GmHsp17.8A-CII | |  |  | | Pt21.8-ER | 823806 |
|  |  | | Glyma04g05720 | GmHsp17.9A-CII | |  |  | | Pt23.1-P | 574673 |
|  |  | | Glyma14g06910 | GmHsp17.9B-CI | |  |  | | Pt23.9-MI | 817274 |
|  |  | | Glyma14g11420 | GmHsp17.9C-CII | |  |  | |  |  |
|  |  | | Glyma17g34220 | GmHsp17.9D-CII | |  |  | |  |  |
|  |  | | Glyma20g01930 | GmHsp17.9E-CII | |  |  | |  |  |
|  |  | | Glyma14g11430 | GmHsp18.0A-CII | |  |  | |  |  |
|  |  | | Glyma16g01440 | GmHsp18.0B-CIII | |  |  | |  |  |
|  |  | | Glyma07g04860 | GmHsp18.2A-CIII | |  |  | |  |  |
|  |  | | Glyma07g32050 | GmHsp18.5A-CI | |  |  | |  |  |
|  |  | | Glyma13g24490 | GmHsp18.5B-CI | |  |  | |  |  |
|  |  | | Glyma07g03450 | GmHsp21.6-MII | |  |  | |  |  |
|  |  | | Glyma20g35650 | GmHsp22.0-ER | |  |  | |  |  |
|  |  | | Glyma13g36220 | GmHsp22.2-CVI | |  |  | |  |  |
|  |  | | Glyma19g01440 | GmHsp22.3B-ER | |  |  | |  |  |
|  |  | | Glyma10g32000 | GmHsp22.4-ER | |  |  | |  |  |
|  |  | | Glyma16g33130 | GmHsp22.5-ER | |  |  | |  |  |
|  |  | | Glyma12g01580 | GmHsp23.8-MI | |  |  | |  |  |
|  |  | | Glyma18g10760 | GmHsp23.9-P | |  |  | |  |  |
|  |  | | Glyma08g43120 | GmHsp25.3-P | |  |  | |  |  |
|  |  | | Glyma08g22630 | GmHsp25.7-MII | |  |  | |  |  |
|  |  | | Glyma04g40790 | GmHsp26.0-P | |  |  | |  |  |
|  |  | | Glyma06g14000 | GmHsp26.1-P | |  |  | |  |  |
|  |  | | Glyma11g37450 | GmHsp27.3-P | |  |  | |  |  |
