# Supplementary material for: Genome-wide analysis of the potato Hsp20 gene family: identification, genomic organization and expression profiles in response to heat stress
Source: BMC Genomics. 2018 Jan 18;19:61. doi: 10.1186/s12864-018-4443-1 (PMC5774091; doi:10.1186/s12864-018-4443-1)
Supplement: Supplementary file 3 — The primer sequences of 48 StHsp20 genes used for qRT-PCR. (DOCX 15 kb) [file 12864_2018_4443_MOESM3_ESM.docx]

| **Gene** | **Forward Primer (5'-3')** | **Reverse Primer (5'-3')** |
| --- | --- | --- |
| StHSP20-1 | TAGTTCTTCCCATTCCAGTGA | TACTTGGCTTATTCAGGTTTG |
| StHSP20-2 | CGGAAATGTGAGTTGTTTGG | TCACGTTGGCGACATAGAGC |
| StHSP20-3 | TAGTCCAAAATCCAATGAACAA | TAGCTTTCTTTGAAGACCTCAT |
| StHSP20-4 | TTCTGGAAAGTTCTGGAGAC | ACCAGCACCATTTACTTGTT |
| StHSP20-5 | AACAACAGAGCCAATAAGG | GCAGCACTAGCACCAAGAT |
| StHSP20-6 | AGGGCTTAGCAAAGAGGATGT | GTTCCACTCTATGCCACTTCA |
| StHSP20-7 | AAATACGCACTCCTTGGG | ATCCGGGAGACTTAAACG |
| StHSP20-8 | TGAACACGACGACGAGACA | CTACCACTGGACCTCCCTC |
| StHSP20-9 | CGCCTTAACTCGATCAGA | TCCAGAATTTCCCACAAG |
| StHSP20-10 | GAAGAAGAGGGATGCAAGTAC | CAGTCTTAGCCTTAGATGGAG |
| StHSP20-11 | GCGGCAAGAAGGACGAAT | TCGAGAAATCGCCACCTC |
| StHSP20-12 | TTCAAGATTCCAGATGGGGTTA | ATTTTCCTCTGCTTTTGGCTTA |
| StHSP20-13 | CGACTGGCGTGAAACTGAT | AGCCCACAACTTATACCTTCC |
| StHSP20-14 | TTTGCCAGGACCTGTTGAT | CTGGGAAAGAGCCATTGTA |
| StHSP20-15 | AGAGGAGGTGCGAGTTCAATC | GCAGAAGTCTGAAGAGGATCG |
| StHSP20-16 | AGGAATGACCAAAGAAGATGT | AATATTCTCAGGCAAAGCAAT |
| StHSP20-17 | AAGAATGATACTTGGCATCGT | TTGGAACAGTGACAGTAAGCA |
| StHSP20-18 | TAAGAATGATAAGTGGCATCG | GTTTCTTCACCTCTTCCTTTG |
| StHSP20-19 | CGATCTTCCAGGGCTTAAA | AATTTCCCACTGCTGCGTT |
| StHSP20-20 | GAAGTGAAAGTGGAGATCGT | CGATGCCACTTATCATTCTT |
| StHSP20-21 | AGAACGATACTTGGCATCGG | AATGGACTTGACATCAGGCT |
| StHSP20-22 | ACTCAGTGTAAAGTGGCATCA | TTAGCATTACTTGGAAGAGGC |
| StHSP20-23 | GTGTTCGGAGGATTGAGCT | TATTTGGAGGGATCTTTGG |
| StHSP20-24 | TGCGAATACTGATGCGATTT | GTTTCTTTGGCTGAGGAGGA |
| StHSP20-25 | TACTGATGCGATTTCTGCTG | GATTTTGGTTTCTTTGGCTC |
| StHSP20-26 | ATGGACATGCCTGGTCTC | TCTCGCCTTCTCCTTTGA |
| StHSP20-27 | ATTTCGTCTCGGACTATCAT | ATTCGAACAGATTTCAATGT |
| StHSP20-28 | CAGTCAGTAGGACCGAAAGAG | CAGTGAAGTAAACCGTGTTGT |
| StHSP20-29 | AGTATTGACGATTAGTGGGAGA | ATAGACGCCTTCGGTTGT |
| StHSP20-30 | GAAAATTACTGGGGAAACAA | TTAACATAATCAGCCCTTGA |
| StHSP20-31 | TTGCCTAAACATCAGGCTACTA | TTCCTTGGCATCTTCAGCTTCT |
| StHSP20-32 | AATGATCAGTGGCACCGTG | CCATCGCTGCCTTTATTTC |
| StHSP20-33 | GAGAATACGAAGACGGGAGA | TTATTGACAAAGGGCCTTTC |
| StHSP20-34 | AGAGGAAGTGAAAGTCGAAGTT | TTTAGGAACAGTCACAGTCAGC |
| StHSP20-35 | GGAAGAAGGAAGGATTTTACAG | TTTTGGAACAGTCACAGTCAGT |
| StHSP20-36 | GAATGATAAGTGGCATCGTGTA | TTTACCTCTTCCTTTGGAACAA |
| StHSP20-37 | CGTGGCAAGTTCTGTAGAAG | AATCTCCTGCTTAGGAATTG |
| StHSP20-38 | GAAGTGAAGGTACAAGTGGAAG | TCCCTTCTTAGGAATAGTGACA |
| StHSP20-39 | GGAGCCAAGAAATCTAGAAC | TCAACGATTCAACTGAACTC |
| StHSP20-40 | ACGTAGATGCGATAAAGGC | ACCACGATAGCTGCAACAC |
| StHSP20-41 | TTTGGAGACAGTTTCGTTTG | TTTTCCTTCTTGTTTGCTCT |
| StHSP20-42 | ACCAGAAGACAAGCCACAAC | CAGCATTGGTAGCCTGATGT |
| StHSP20-43 | AGTTTGTAAGGAGGTTTAGGTT | AGGAACAGTTACAGTAAGCACT |
| StHSP20-44 | GAGAATGTGAAAATGGAGGAAA | ATTAATAGCTTTCACCTCAGGC |
| StHSP20-45 | GGTTGCTGAGCCAATCGTA | CCAGGCACCAGTGCATAGA |
| StHSP20-46 | AATTACAAAATCCGTTACGAC | ACTTCTTGATGACCAAAACTC |
| StHSP20-47 | AGCGAGATTTCAAGAAGACG | CGGGCAATTCATTATTAGCA |
| StHSP20-48 | GACAATCTTTGGGGTGCTACT | GGCACATGAACATGAAGACAC |
