# Supplementary figures and images for: Genome-wide analysis of the potato Hsp20 gene family: identification, genomic organization and expression profiles in response to heat stress
Source: BMC Genomics. 2018 Jan 18;19:61. doi: 10.1186/s12864-018-4443-1 (PMC5774091; doi:10.1186/s12864-018-4443-1)

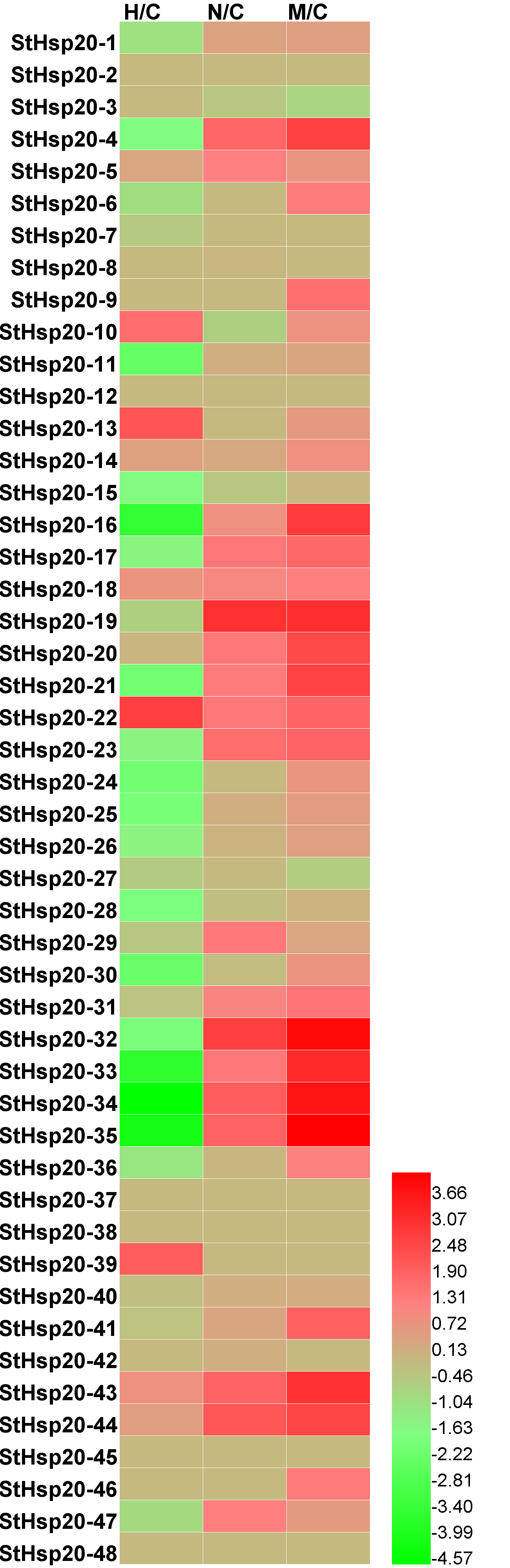

Supplement: Supplementary file 4 — Heatmap of StHsp20s under heat, salt and drought stress. (TIFF 327 kb) [file 12864_2018_4443_MOESM4_ESM.tif]
